# Growth Differentiation Factor 11 Is a Circulating Regulator of Oligodendrocyte Differentiation and CNS Myelin Formation and Repair

**DOI:** 10.1002/cns.71000

**Published:** 2026-06-22

**Authors:** Zhen Zhang, Min Yan, Wenjiao Huang, Yongchang Chen, Tao Lv, Wenli Chen

**Affiliations:** ^1^ State Key Laboratory of Primate Biomedical Research, Institute of Primate Translational Medicine Kunming University of Science and Technology Kunming China; ^2^ Neurology Department, The First People's Hospital of Yunnan Province The Affiliated Hospital of Kunming University of Science and Technology Kunming China; ^3^ Medical School Kunming University of Science and Technology Kunming China

**Keywords:** demyelination, growth differentiation factor 11, multiple sclerosis, myelination, oligodendrocyte, oligodendrocyte progenitor cells

## Abstract

**Aims:**

Remyelination failure is a major contributor to progressive neurological disability in multiple sclerosis (MS). Although oligodendrocyte progenitor cells (OPCs) persist in demyelinated lesions, they often fail to differentiate into myelinating oligodendrocytes. This study aimed to determine whether growth differentiation factor 11 (GDF11), a circulating member of the TGF‐β superfamily, regulates oligodendrocyte differentiation, CNS myelination, and remyelination.

**Methods:**

The effects of GDF11 were examined using purified mouse OPC cultures, a neonatal developmental myelination model, and two demyelination models: cuprizone‐induced demyelination and experimental autoimmune encephalomyelitis (EAE). OPC differentiation was assessed by immunocytochemistry and morphological analysis. In vivo myelination and remyelination were evaluated using histological, ultrastructural, and behavioral approaches following systemic GDF11 administration.

**Results:**

GDF11 directly promoted OPC differentiation and maturation in vitro without affecting proliferation. Systemic GDF11 enhanced developmental myelination in neonatal mice, increasing myelin gene expression, myelinated axon density, and myelin thickness. Although GDF11 did not prevent active demyelination, it significantly accelerated remyelination and functional recovery following cuprizone withdrawal and in the EAE model, and this was accompanied by enhanced oligodendrocyte differentiation and reduced neuroinflammation.

**Conclusion:**

These findings identify GDF11 as a circulating regulator of oligodendrocyte maturation and CNS myelin formation and repair, highlighting its therapeutic potential for demyelinating diseases.

## Graphical abstract

Systemically administered GDF11 enhances CNS myelin formation and repair across both developmental and disease contexts. GDF11 promotes the differentiation of oligodendrocyte progenitor cells into mature oligodendrocytes, increases myelin sheath thickness in neonatal mice, and accelerates remyelination in both the cuprizone‐induced demyelination model and the experimental autoimmune encephalomyelitis model. These myelin‐restorative effects are accompanied by a marked improvement in motor function.

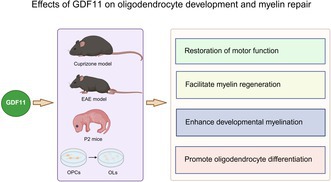

## 1 Introduction

Multiple sclerosis (MS) is a chronic demyelinating disease of the central nervous system (CNS) characterized by aberrant autoimmune response, oligodendrocyte (OL) loss, demyelination, and progressive neurodegeneration [1]. Although current disease‐modifying therapies effectively suppress CNS inflammation and reduce relapse rates, they largely fail to prevent long‐term disease progression and irreversible neurological disability [2, 3, 4]. This limitation is primarily due to the failure of these therapies to promote efficient endogenous remyelination within the adult CNS [5, 6]. Notably, oligodendrocyte progenitor cells (OPCs) persist within demyelinated MS lesions but frequently fail to differentiate into myelinating OLs. Thus, impaired OPC differentiation represents a key pathological bottleneck and a promising therapeutic target.

Remyelination is a highly regulated, multistep process that is profoundly influenced by the local CNS microenvironment. A range of growth factors, including platelet‐derived growth factor (PDGF), basic fibroblast growth factor (bFGF), and brain‐derived neurotrophic factor (BDNF), have been shown to enhance OPC proliferation and survival [7, 8, 9, 10]. Other peripherally derived molecules, such as fibroblast growth factor 21 (FGF21) and prostacyclin, promote remyelination through endocrine signaling [11, 12, 13]. Among these signaling pathways, ligands of the transforming growth factor‐β (TGF‐β) superfamily—including TGF‐β1 and Activin A—play pivotal roles in OL development and remyelination by activating canonical Smad2/3‐dependent and non‐canonical MAPK signaling pathways [14, 15, 16, 17, 18].

Growth differentiation factors (GDFs) represent a distinct subgroup within the TGF‐β superfamily; however, unlike the well‐studied TGF‐β ligands, their functions in CNS myelination and remyelination remain poorly defined. Among them, growth differentiation factor 11 (GDF11) has attracted increasing attention due to its pleiotropic roles in CNS development, adult neurogenesis, vascular remodeling, and age‐related functional rejuvenation [19, 20, 21, 22, 23, 24, 25, 26]. Mechanistically, GDF11 signals through activin receptor‐like kinases ALK4, ALK5, and ALK7 to activate Smad2/3 signaling—pathways previously implicated in promoting OL differentiation and maturation [27]. These observations raise the intriguing possibility that GDF11 may act as a circulating factor capable of influencing oligodendroglial lineage progression and CNS myelin dynamics.

To test this hypothesis, we systematically examined the effects of peripherally administered GDF11 on OPC differentiation, developmental myelination, and remyelination in multiple experimental paradigms. We demonstrate that GDF11 directly promotes OPC maturation in vitro, enhances developmental myelination in neonatal mice, and markedly accelerates remyelination and functional recovery in both toxic and autoimmune demyelination models. Together, our findings identify GDF11 as a previously unrecognized systemic regulator of CNS myelin formation and repair.

## 2 Materials and Methods

### 2.1 Animals

C57BL/6J mice were housed in a specific pathogen‐free (SPF) facility under controlled conditions (22°C ± 2°C, 50%–60% humidity) with a 12‐h light/dark cycle. Mice had free access to autoclaved standard chow and water. Both male and female mice were used as specified in each experimental section. All animal experiments were conducted in accordance with the guidelines of the National Institutes of Health Guide for the Care and Use of Laboratory Animals.

### 2.2 Isolation and Culture of Mouse OPCs

OPCs were isolated from postnatal day 1 (P1) C57BL/6J mouse brains using an immunopanning method with minor modifications based on a previously published protocol [28]. Briefly, culture dishes were coated with anti‐mouse IgM (10 μg/mL; Sigma, SAB5600199), followed by incubation with A2B5 antibody (Invitrogen, MA1‐90445; 1:200). Cortical tissue was digested with trypsin–EDTA (Gibco, 25200072; 1:2.5), and the cell suspension was incubated on the coated dishes. After removing non‐adherent cells, bound A2B5^+^ OPCs were detached and cultured in DMEM/F12 medium (Gibco, 11960044) supplemented with N2 (Gibco, 17502‐048), 20 ng/mL platelet‐derived growth factor‐AA (PDGF‐AA; PeproTech, 100‐13), 20 ng/mL basic fibroblast growth factor (bFGF; PeproTech, 100‐18B), 5 ng/mL neurotrophin‐3 (NT‐3; R&D, 267‐N3), 10 ng/mL ciliary neurotrophic factor (CNTF; PeproTech, 450‐13), and 2 mM L‐glutamine (Gibco, 25030‐018). To assess culture purity, freshly isolated cells were subjected to immunofluorescence staining for Olig2, and the percentage of Olig2‐positive cells was quantified. Purified OPCs (150,000 cells per 25 cm^2^ flask) were cultured for an average of 4 days until reaching approximately 80% confluence, at which point they were passaged once (passage 1, P1) to avoid contact inhibition and maintain proliferative capacity. All subsequent proliferation and differentiation assays were performed using P1 cells.

### 2.3 OPC Proliferation Assays

OPC proliferation was evaluated using the MTT assay (Roche, 11465007001). Cells were seeded in 96‐well plates at 5 × 10^3^ cells per well and allowed to adhere overnight. After attachment, cells were treated with vehicle (sterile saline) or 100 ng/mL recombinant human GDF11 (Origene, Cat# TP72907) in OPC proliferation medium for 48 h. Each treatment group contained six technical replicate wells per plate. Following treatment, 10 μL of MTT reagent was added to each well and incubated for 4 h at 37°C. Subsequently, 100 μL of solubilization solution was added and incubated overnight at room temperature in the dark. Absorbance was measured at 562 nm with a reference wavelength of 650 nm using a microplate reader. Three independent biological replicates (separate OPC isolations) were performed. For each independent experiment, the mean absorbance of the six technical replicates was calculated, and the vehicle‐treated group was set to 100%. Results are expressed as mean ± SEM from three independent experiments.

### 2.4 OPC Differentiation Assays

Purified OPCs were seeded at a density of 20,000 cells per well onto poly‐L‐lysine‐coated 12 mm glass coverslips (Corning, 354085) in 24‐well plates. Cells were allowed to adhere for 2 h in differentiation medium consisting of DMEM/F12 supplemented with N2, 30 ng/mL triiodothyronine (T3; Sigma‐Aldrich, T2877), 5 ng/mL NT‐3, 10 ng/mL CNTF, and 2 mM L‐glutamine. Recombinant human GDF11 (dissolved in sterile saline) was then added to the medium at the indicated concentrations. For gene expression analysis, cells were incubated for 5 days, and total RNA was extracted using TRIzol reagent (Invitrogen, 15596026CN) following the manufacturer's protocol. For immunocytochemistry, cells were fixed at specific time points as described below.

### 2.5 Immunocytochemistry

For surface antigen detection on live OPCs, cells were incubated with antibodies against O4 (1:200, R&D Systems, MAB1326) or O1 (1:200, R&D Systems, MAB1327) in blocking buffer (10% goat serum in DMEM medium) for 30 min, washed three times with DMEM medium, and then incubated with secondary antibody (goat anti‐mouse IgM Alexa Fluor 594, 1:500; Invitrogen, A21044) in blocking buffer (10% goat serum in DMEM medium) for 30 min. After washing three times with DMEM, cells were fixed with 4% PFA for 10 min, washed with PBS, stained with DAPI solution (Sigma‐Aldrich, D9524), and mounted with mounting medium. For intracellular protein staining, cells were fixed with 4% PFA, washed with PBS, and incubated with blocking buffer (0.1% Triton X‐100, 10% goat serum in PBS) for 30 min. Subsequently, cells were incubated with primary antibodies against MBP (Covance, SMI‐99P, 1:250) and Olig2 (Proteintech, 25754‐1‐AP, 1:200) for 1 h, washed with PBS, and then incubated with Alexa Fluor 594‐conjugated secondary antibodies (Invitrogen, A‐11012, 1:1000) for 1 h. After washing with PBS, cells were stained with DAPI solution and mounted with mounting medium. Images were acquired using a Nikon AXR confocal microscope. For analysis of the proportion of O4^+^, O1^+^, or MBP^+^ cells relative to the total number of DAPI‐stained nuclei, three independent experiments were performed. In each experiment, one coverslip per condition was used, and five random microscopic fields per coverslip were analyzed. Data from the five fields were then used for statistical analysis. For quantification of OL morphological complexity, only MBP‐positive cells displaying visible membrane structures were quantified. The MBP^+^ membrane sheet area was assessed from three independent experiments, each consisting of one coverslip per condition and five random microscopic fields per coverslip. A total of ≥ 30 cells per condition from three independent cultures were measured using ImageJ software (National Institutes of Health, USA).

### 2.6 Developmental Myelination

To investigate the effect of GDF11 on developmental myelination, C57BL/6J mouse pups were administered GDF11 (1 mg/kg in sterile saline) or vehicle (sterile saline) once daily via intraperitoneal injection from postnatal day (P) 2 to P16. On P8, P16, and P42, mice were anesthetized with 2% isoflurane (RWD, R510‐22‐10) or Avertin. For protein and mRNA analysis, mice were transcardially perfused with ice‐cold phosphate‐buffered saline (PBS, Sangon, B548117), and then the brains were removed. The brains were coronally sectioned into 1‐mm thick slices using a mouse brain matrix. Under a dissecting microscope, the corpus callosum was dissected from each hemisphere (including both the medial and lateral portions, from the genu to the splenium). Tissues were immediately frozen in liquid nitrogen and stored at −80°C until use. For histochemistry, mice were transcardially perfused with ice‐cold PBS to remove blood, followed by perfusion with 4% paraformaldehyde (PFA). Brains were dissected, post‐fixed in 4% PFA overnight at 4°C, cryoprotected in 30% sucrose, and embedded in OCT compound. Coronal sections (20 μm thick) were cut and processed for MBP immunohistochemistry. For ultrastructural analysis, mice were deeply anesthetized with Avertin and transcardially perfused with ice‐cold PBS, followed by perfusion with electron microscopy fixative containing 4% paraformaldehyde and 2% glutaraldehyde in 0.1 M sodium cacodylate buffer (pH 7.2). Brains were removed and coronally sliced into 1‐mm thick sections. The middle region of the corpus callosum was dissected under a dissecting microscope, post‐fixed in the same fixative, and processed for transmission electron microscopy.

### 2.7 Cuprizone‐Induced Demyelination Model

Twelve‐week‐old male C57BL/6J mice were fed a standard rodent chow containing 0.2% (w/w) cuprizone (Sigma‐Aldrich, C9012) for 5 weeks to induce demyelination. Age‐matched control mice received normal chow. To assess the protective effect of GDF11 during active demyelination, a cohort of mice received daily intraperitoneal injections of recombinant human GDF11 (1 mg/kg; Origene, TP726907) or vehicle throughout the 5‐week cuprizone feeding period (*n* = 12 per group). Body weight was measured twice per week, and rotarod behavioral tests were performed at week 5. To assess remyelination, another cohort received GDF11 or vehicle for 2 weeks after cuprizone withdrawal. Rotarod tests were performed 2 weeks after withdrawal. At the experimental endpoint, tissue collection and processing for mRNA analysis and histochemistry (Luxol fast blue, MBP, Iba1) were performed as described in the “Developmental myelination” section. For ultrastructural analysis, the lateral corpus callosum was dissected for transmission electron microscopy.

### 2.8 Experimental Autoimmune Encephalomyelitis (EAE) Model

EAE was induced in 10‐week‐old female C57BL/6J mice via subcutaneous immunization with MOG_35–55_/CFA emulsion, followed by pertussis toxin injections (Hooke Laboratories, EK‐2110). Mice were monitored daily and clinical scores (0–5 scale: 0, no clinical signs; 1, limp tail; 2, limp tail and hind limb weakness; 3, complete hind limb paralysis; 4, hind limb paralysis with partial forelimb paralysis; and 5, moribund or death) were recorded. On day 17 post‐immunization, mice with a score of 3 were randomly assigned to receive daily intraperitoneal injections of either GDF11 (1 mg/kg) or vehicle for two weeks. All treatments and clinical assessments were performed in a blinded manner. At the experimental endpoint, processing of spinal cord for ultrastructural analysis and histochemistry (Luxol fast blue, MBP) was performed as described in the “Developmental myelination” section. For ultrastructural analysis, the ventral white matter of the lumbar spinal cord was processed for transmission electron microscopy.

### 2.9 Rotarod Test

Motor performance was evaluated using an accelerating rotarod apparatus (Ugo Basile). On the testing day, mice were kept in their home cages and acclimated to the testing room for at least 20 min before the experiment. Mice were trained on the rod at a constant speed of 4 rpm for 1 min. During testing, the rod accelerated from 4 to 40 rpm over a 5‐min period. The latency to fall was recorded, with a maximum cutoff time of 300 s. Each mouse underwent three trials per session, with intervals of at least 15 min between trials. The average latency across the three trials was calculated for statistical analysis.

### 2.10 Immunofluorescence Staining

Frozen tissue sections (20 μm thickness) were incubated in blocking buffer (PBS containing 0.2% Triton X‐100 and 5% normal goat serum) for 1 h at room temperature. Sections were then incubated overnight at 4°C with primary antibodies against MBP (Covance, SMI‐99P, 1:250) and Iba1 (HUABIO, ET1705‐78, 1:200). After washing three times with PBS, sections were incubated with Alexa Fluor 594‐conjugated secondary antibodies (Invitrogen, A‐11012, 1:1000) for 1 h at room temperature. Sections were then washed, counterstained with DAPI solution, and mounted with mounting medium. Images were acquired using a Leica DM4B microscope with consistent exposure settings applied for quantification.

### 2.11 Luxol Fast Blue (LFB) Staining

LFB staining was performed on lumbar spinal cord sections to evaluate demyelination. After warming to room temperature, sections were sequentially washed with PBS, 70% and 95% alcohol, then stained with 0.1% LFB solution (Sigma‐Aldrich, L0924) at 56°C overnight. Following staining, sections were rinsed in 95% ethanol to remove excess dye and differentiated in 0.05% lithium carbonate (Sigma, 255823) solution for 30 s. This was followed by differentiation in 70% ethanol for another 30 s until gray and white matter could be clearly distinguished. Following a distilled water wash, sections were dehydrated through a graded alcohol series (70%, 95%, 100%), cleared in xylene, and mounted. Images were acquired using a Leica DM4B microscope.

### 2.12 Western Blot

Protein concentrations were determined by BCA assay. Equal amounts of protein (20–30 μg) were separated by SDS‐PAGE, transferred to PVDF membranes (Millipore, HVLP14250), and blocked with 5% non‐fat milk. Membranes were incubated overnight with primary antibodies (MBP, Covance, SMI‐99P, 1:1000; GAPDH, Proteintech, # 60004‐1‐Ig, 1:1000) at 4°C, followed by HRP‐conjugated secondary antibody (1:10,000; Jackson ImmunoResearch) for 1 h. Bands were visualized with ECL (Biosharp, BL520B), and densitometric analysis was performed using ImageJ software (National Institutes of Health, USA).

### 2.13 qRT‐PCR

Total RNA was extracted from brain/spinal cord tissue with TRIzol, and its quality was assessed. Reverse transcription was performed using 1 μg RNA. Quantitative PCR was conducted with SYBR Green Master Mix (Promega, A2791) on an ABI 7300 system (95°C for 2 min; 40 cycles: 95°C for 15 s, 60°C for 1 min), including melt curve analysis. Reactions were run in triplicate. mRNA levels were normalized to β‐actin and calculated using the 2^−ΔΔCt^ method. The sequences of the primers used are as follows: *Cnp*‐F: AAGGCCTTGCCATACGATCT; *Cnp*‐R: CGCTGGGGCAGAAGAATAC; *Mbp*‐F: AGCCCTCTGCCCTCTCAT; *Mbp*‐R: GGTAGTTCTCGTGTGTGAGTCCT; *Mog*‐F: CTTCTTCAGAGACCACTCTTACCA; *Mog*‐R: GTTGACCCAATAGAAGGGATCTT; *Actb*‐F: CTCTTCCAGCCTTCCTTCCT; *Actb*‐R: TGTTGGCGTACAGGTCTTTG; *Plp1*‐F: TGCTCGGCTGTACCTGTGTACATT; *Plp1*‐R: TACATTCTGGCATCAGCGCAGAGA; *Il1b*‐F:GTATGGGCTGGACTGTTTC; *Il1b*‐R: GCTGTCTGCTCATTCACG; *Tnf*‐F: TAGTCCTTCCTACCCCAATTTCC; *Tnf*‐R: TTGGTCCTTAGCCACTCCTTC.

### 2.14 Transmission Electron Microscopy (TEM)

Brain and spinal cord samples were post‐fixed, treated with 1% osmium tetroxide, and dehydrated through an ethanol series (30%, 50%, 70%, 90%, and 100%), followed by two washes in propylene oxide for 10 min. Samples were infiltrated in a mixture of propylene oxide and Embed‐812 resin (Electron Microscopy Sciences, Hatfield, PA) with increasing resin concentration, and finally embedded in pure resin. Blocks were polymerized at 60°C for 48 h. Ultrathin sections were cut using a Leica EM UC7 ultramicrotome (Leica Microsystems), mounted on copper grids, and stained with 2% uranyl acetate for 15 min followed by lead citrate for 10 min. Sections were imaged using a transmission electron microscope (JEM‐1400, JEOL) operated at 120 kV. For TEM‐based analyses of myelination, the percentage of myelinated axons and g‐ratios were calculated from high‐magnification images taken from the ventral spinal cord and the corpus callosum. For g‐ratio analysis, at least 100 axons from three mice per group were quantified using ImageJ software (National Institutes of Health, USA).

### 2.15 In Situ Hybridization

A 969‐bp probe targeting proteolipid protein (PLP) was generated by PCR using primers derived from the Allen Brain Atlas and flanked by T7/T3 promoter sequences. PCR was performed using cDNA synthesized from isolated RNA, followed by re‐amplification and verification through sequencing. Digoxigenin‐labeled RNA probes were transcribed using the Ambion Maxiscript Transcription Kit (Invitrogen) with digoxigenin‐11‐UTP (Roche), employing T3 RNA polymerase for sense control probes and T7 for antisense probes. In situ hybridization was carried out according to a previously described standard protocol. Probes were diluted 1:50 in hybridization buffer and hybridized overnight at 65°C. PLP^+^ mature OL in the corpus callosum were quantified by counting positive cells across three sections per animal using ImageJ software.

### 2.16 Statistical Analysis

All statistical analyses were conducted using GraphPad Prism version 8.4.3. Data are presented as mean ± SEM. Group comparisons were made using unpaired two‐tailed Student's *t*‐tests or one‐way/two‐way analysis of variance (ANOVA) followed by Tukey's or Sidak's post hoc tests. All quantifications were performed by observers blinded to treatment conditions. A *p*‐value of less than 0.05 was considered statistically significant.

## 3 Results

### 3.1 GDF11 Directly Promotes OPC Differentiation and Maturation In Vitro

**FIGURE 1:**
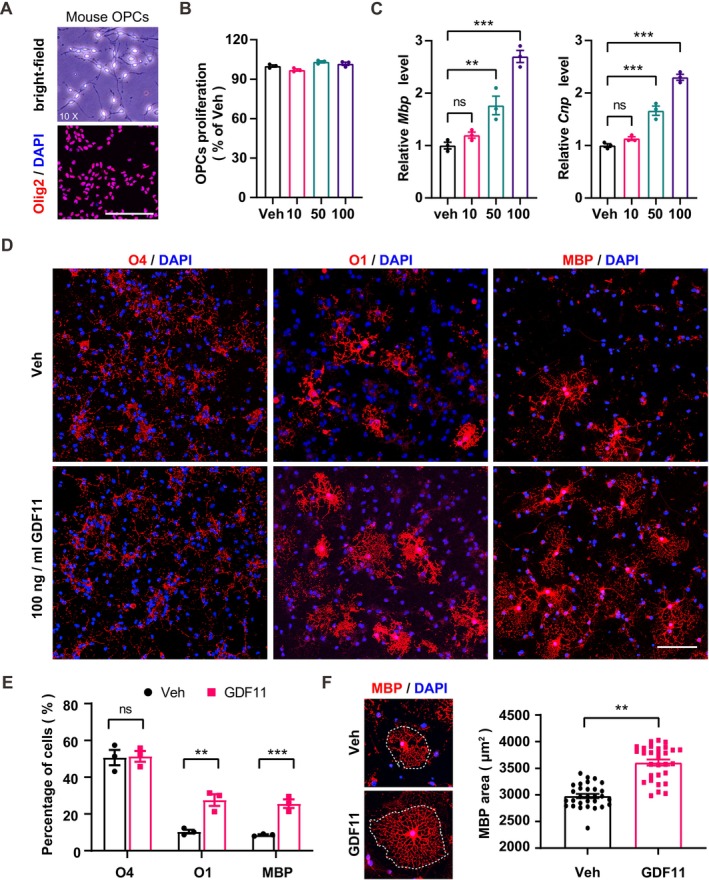
GDF11 promotes OPC differentiation and maturation in vitro. (A) Representative bright‐field image of purified OPCs from mouse pup brains and immunofluorescence staining of Olig2 (red) with DAPI (blue) for purity assessment. Scale bar, 100 μm. (B) OPCs were treated with vehicle (Veh) or GDF11 for 48 h, and cell viability was assessed by MTT assay. Data are presented as mean ± SEM from three independent experiments (each with six technical replicates per group). (C) Real‐time PCR analysis of *Mbp* and *Cnp* mRNA levels in OPC cultures after GDF11 or Veh treatment (*n* = 3 independent experiments). (D) Representative immunofluorescence images of OPC differentiation after treatment with GDF11 (100 ng/mL) or Veh. Scale bar, 100 μm. (E) Quantification of the relative proportion of maturing OLs after GDF11 (100 ng/mL) or Veh treatment (*n* = 3 independent experiments). (F) Representative immunofluorescence images of MBP (red) showing membrane sheet area (outlined by dashed boxes) and quantification of MBP^+^ membrane sheet area after GDF11 (100 ng/mL) or Veh treatment (*n* = 3 independent experiments). The data are presented as mean ± SEM. **p < 0.01, ***p < 0.001; ns, not significant.

To determine whether GDF11 exerts direct effects on oligodendrocyte lineage cells, we isolated and purified mouse OPCs using an A2B5‐based immunopanning method. The majority of purified cells exhibited typical OPC morphology (Figure 1A), characterized by small cell bodies and branched processes as previously described [29, 30]. Immunofluorescence staining revealed that > 95% of the cells expressed the oligodendrocyte lineage marker Olig2 (Figure 1A). OPCs were then treated with recombinant GDF11 at 10, 50, and 100 ng/mL. MTT assays showed no significant differences in cell viability or proliferation between any GDF11‐treated group and the vehicle (Veh)‐treated OPCs (Figure 1B), indicating that GDF11 does not affect OPC expansion.

We next examined the effects of GDF11 on OPC differentiation. Following five days of differentiation in the presence of GDF11, qRT‐PCR analysis revealed a robust, dose‐dependent upregulation of the myelin‐associated genes *M*bp and *Cnp* (Figure 1C). To further characterize lineage progression, OPCs were differentiated on coverslips and immunostained for stage‐specific markers at defined time points. While GDF11 (100 ng/mL) did not significantly alter the proportion of O4^+^ pre‐oligodendrocytes at day 2, it significantly increased the proportion of O1^+^ immature OLs at day 4 and MBP^+^ mature OLs at day 6 (Figure 1D,E). In addition to promoting OL maturation, GDF11 markedly enhanced morphological complexity. Quantitative analysis demonstrated a significant increase in the MBP^+^ membrane sheet area following 100 ng/mL GDF11 treatment (Figure 1F), consistent with enhanced membrane elaboration required for myelin formation. Together, these data demonstrate that GDF11 acts directly on OPCs to promote their differentiation and functional maturation into myelinating OLs.

### 3.2 Systemic GDF11 Enhances Developmental Myelination In Vivo

**FIGURE 2:**
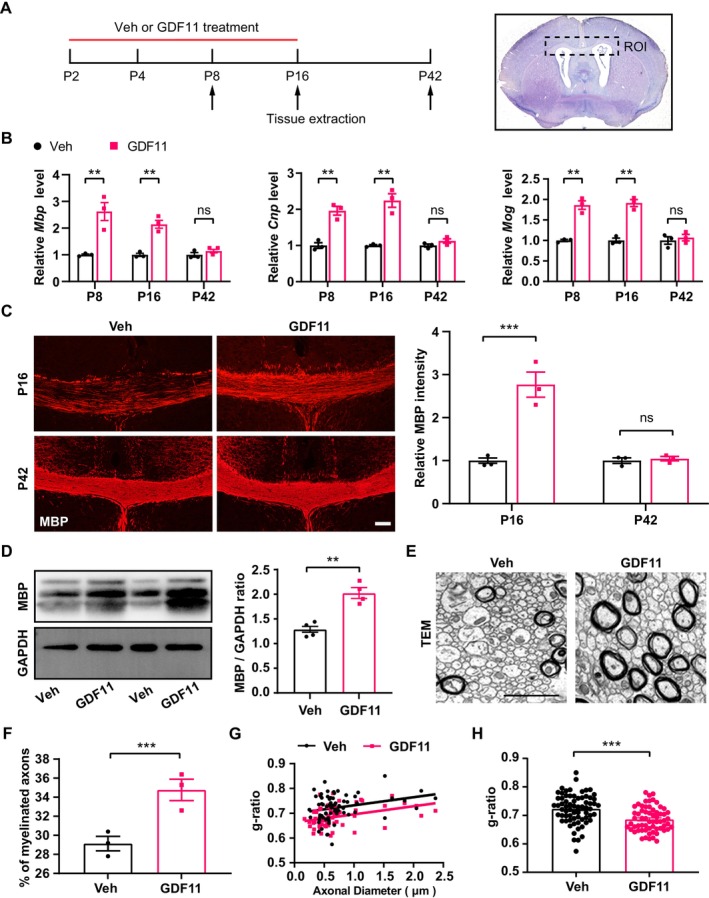
GDF11 enhances myelination in vivo. (A) Timeline of GDF11 or Veh administration during early postnatal development and experimental sampling. The schematic coronal brain section shows the region of interest (ROI, corpus callosum) outlined by the dashed box, which was dissected for subsequent analyses. (B) Real‐time PCR analysis of *Mbp, Cnp*, and *Mog* mRNA levels in the corpus callosum at P8, P16, and P42 (*n* = 3 mice per group). (C) Representative immunofluorescence staining of MBP in the medial corpus callosum of Veh‐ and GDF11‐treated mice at P16 and P42, and quantification of relative MBP fluorescence intensity. Scale bar, 100 μm. (D) Western blot analysis of MBP expression in the corpus callosum at P16, and quantification of relative MBP protein levels (*n* = 4 mice per group). (E) Transmission electron microscopy (TEM) images of the medial corpus callosum at P16. Scale bar, 2 μm. (F) Percentage of myelinated axons in the corpus callosum at P16 (*n* = 3 mice per group). (G) Scatter plot of myelin g‐ratio versus axonal diameter in the corpus callosum at P16 (> 100 axons per group). (H) Mean g‐ratio in the corpus callosum at P16 (> 100 axons per group). Data are presented as mean ± SEM. **p < 0.01, ***p < 0.001; ns, not significant.

Having established that GDF11 directly promotes OPC differentiation and morphological maturation in vitro, we next asked whether systemic GDF11 administration could enhance myelination in vivo under physiological conditions, where the blood–brain barrier (BBB) remains intact. Neonatal mice (postnatal day 2, P2) were treated daily with systemic injections of GDF11 or vehicle from P2 to P16 (14 consecutive days). Myelination in the corpus callosum was analyzed at P8 (after 6 days of treatment), P16 (at the end of treatment), and P42 (4 weeks after the last injection) (Figure 2A).

qRT‐PCR analysis of the corpus callosum at P8 and P16 revealed significantly elevated expression of key myelin genes (*Mbp*, *Cnp*, and *Mog*) in GDF11‐treated mice compared with Veh‐treated mice (Figure 2B). However, at P42, no differences in the mRNA levels of these myelin genes were observed between the two groups (Figure 2B). Consistent with these findings, immunofluorescence analysis showed a significantly larger MBP^+^ myelinated area within the corpus callosum of GDF11‐treated animals at P16, but at P42, no difference in MBP^+^ area was detected between the groups (Figure 2C). Western blot analysis further confirmed increased levels of major MBP protein isoforms in the corpus callosum after GDF11 treatment at P16 (Figure 2D). Ultrastructural examination by transmission electron microscopy at P16 revealed a higher density of myelinated axons in the medial corpus callosum of GDF11‐treated mice (Figure 2E,F). Moreover, g‐ratio analysis demonstrated a significant reduction in g‐ratio in GDF11‐treated mice compared with Veh‐treated mice across axons of varying calibers, indicating increased myelin thickness (Figure 2G,H).

Collectively, these data demonstrate that systemic GDF11 administration transiently accelerates developmental CNS myelination despite an intact BBB. The enhancement of myelination is evident at P16 but does not persist to P42, suggesting that peripheral GDF11 temporarily promotes myelin formation without altering the final steady‐state myelin content.

### 3.3 GDF11 Promotes Remyelination and Functional Recovery After Cuprizone‐Induced Demyelination

**FIGURE 3:**
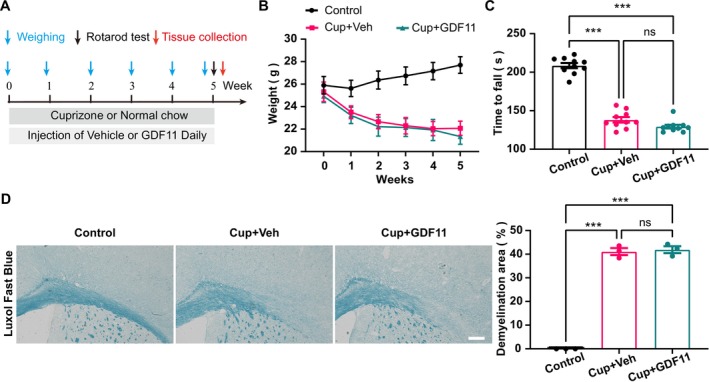
GDF11 does not protect against cuprizone‐induced demyelination or functional impairment. (A) Experimental timeline: Mice were fed cuprizone (Cup) or normal chow (Control) for 5 weeks, with daily GDF11 or Veh administration during this period. (B) Body weight changes of Control (no cuprizone), Cup+Veh, and Cup+GDF11 mice during the 5‐week feeding period (*n* = 10 mice per group). (C) Rotarod performance at week 5 in Control, Cup+Veh, and Cup+GDF11 mice (*n* = 10 mice per group). (D) Representative Luxol fast blue staining images and quantification of demyelinated area in the corpus callosum of Control, Cup+Veh, and Cup+GDF11 mice after 5 weeks of Cup diet (*n* = 3 mice per group). Scale bar, 100 μm. Data are presented as mean ± SEM. ***p < 0.001; ns, not significant.

We next examined whether GDF11 could protect against active demyelination in the cuprizone model. Mice received daily intraperitoneal injections of GDF11 or Veh throughout a 5‐week cuprizone diet (Figure 3A). As expected, cuprizone feeding induced significant body weight loss and motor coordination deficits, as assessed by the rotarod test; however, GDF11 treatment did not prevent these impairments (Figure 3B,C). Consistent with previous reports that cuprizone‐induced demyelination exhibits regional heterogeneity in the corpus callosum [31], Luxol fast blue staining revealed more severe demyelination in the lateral region than in the medial region (Figure 3D). Notably, GDF11 did not reduce cuprizone‐induced demyelination (Figure 3D). These findings indicate that GDF11 does not confer protection against ongoing demyelination.

**FIGURE 4:**
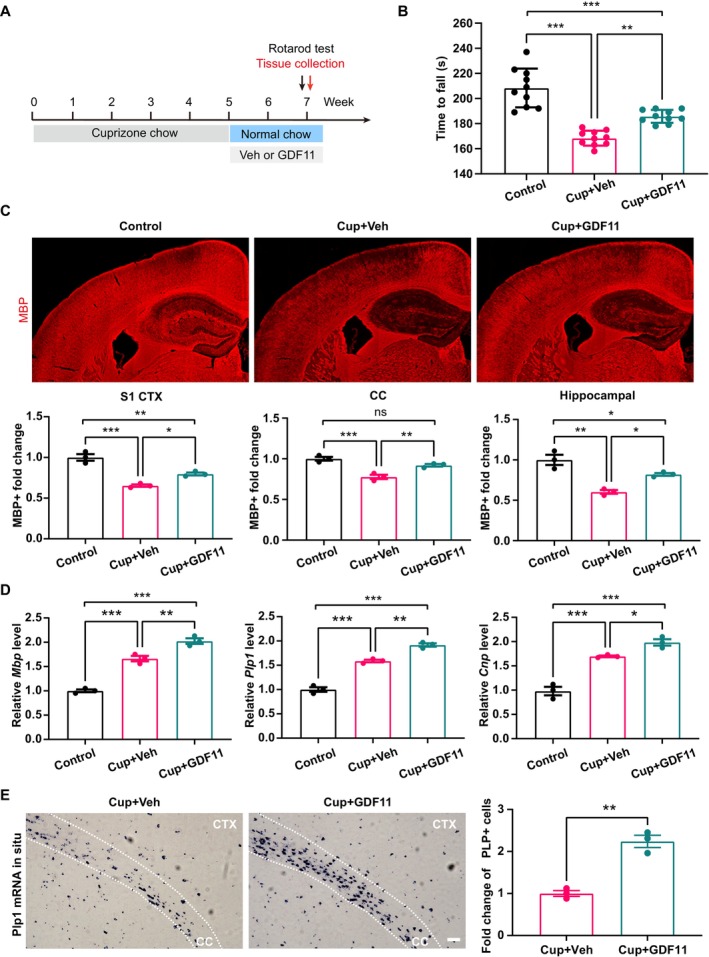
GDF11 promotes functional recovery and remyelination during the repair phase after cuprizone withdrawal. (A) Experimental timeline: mice were fed Cup for 5 weeks followed by 2 weeks of recovery with GDF11 or Veh treatment. A separate group of control mice received normal chow without cuprizone. (B) Rotarod performance (latency to fall) of Control (no cuprizone), Cup+Veh, and Cup+GDF11 mice at the end of the 2‐week recovery period (*n* = 10 mice per group). (C) Representative MBP staining images and quantification of MBP‐positive area (fold change) in the primary somatosensory cortex (S1), corpus callosum (CC), and hippocampus of Control, Cup+Veh, and Cup+GDF11 mice (*n* = 3 mice per group). (D) qRT‐PCR analysis of myelin gene (*Mbp, Plp1, Cnp*) mRNA levels in the CC of Control, Cup+Veh, and Cup+GDF11 mice (*n* = 3 mice per group). (E) Representative images and quantification of PLP^+^ oligodendrocyte number in the lateral CC of GDF11‐ or Veh‐treated mice, detected by in situ hybridization (*n* = 3 mice per group). Scale bar, 100 μm. Data are presented as mean ± SEM. *p < 0.05, **p < 0.01, ***p < 0.001; ns, not significant. CTX, Cortex.

**FIGURE 5:**
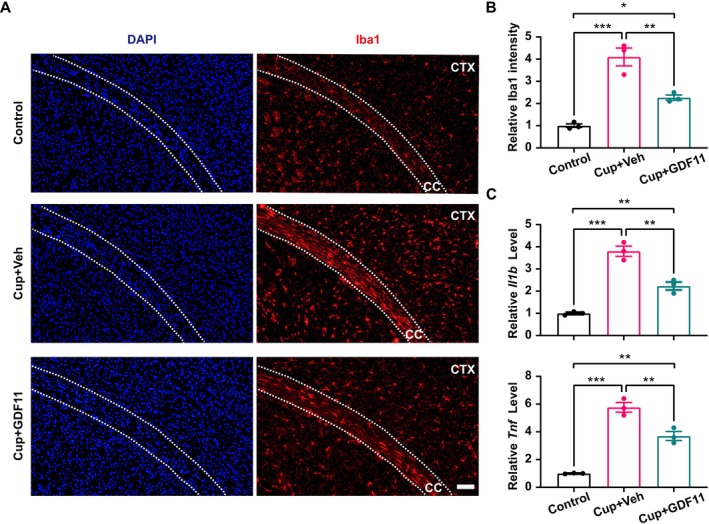
GDF11 attenuates neuroinflammatory responses during remyelination. (A, B) Representative Iba1 immunofluorescence images (A) and quantification (B) of Iba1^+^ microglial density in the lateral corpus callosum (CC) of Control (no cuprizone), Cup+Veh, and Cup+GDF11 mice (*n* = 3 mice per group). Scale bar, 100 μm. (C) qRT‐PCR analysis of pro‐inflammatory cytokine (IL‐1β and TNF‐α) mRNA levels in the CC of the same groups (*n* = 3 mice per group). Data are presented as mean ± SEM. *p < 0.05, **p < 0.01, ***p < 0.001. CTX, cortex.

We next investigated whether GDF11 could enhance remyelination after demyelination had been established. Following 5 weeks of cuprizone exposure, mice were returned to a normal diet and treated daily with GDF11 or Veh for 2 weeks (Figure 4A). GDF11‐treated mice exhibited significantly improved motor recovery on the rotarod compared with Cup+Veh mice (Figure 4B). Histological analysis revealed a marked increase in MBP^+^ myelin area within the primary somatosensory cortex (S1), corpus callosum, and hippocampus of Cup+GDF11 mice relative to Cup+Veh mice (Figure 4C). qRT‐PCR analysis of the corpus callosum showed that cuprizone withdrawal induced spontaneous remyelination in Veh‐treated mice, leading to increased myelin gene expression (*Mbp, Plp1*, and *Cnp*) compared with normal (non‐cuprizone) Control mice (Figure 4D), consistent with previous reports that endogenous repair elevates myelin transcripts during the remyelination phase [32, 33]. Furthermore, GDF11 treatment further upregulated these myelin genes compared with Cup+Veh mice (Figure 4D). In situ hybridization further confirmed an increased number of PLP^+^OLs in the lateral parts of the corpus callosum of Cup+GDF11 mice (Figure 4E), indicating enhanced OL differentiation. In parallel, GDF11 treatment attenuated neuroinflammatory responses. The density of Iba1^+^ microglia was significantly reduced in GDF11‐treated mice compared with Cup+Veh mice (Figure 5A,B), accompanied by decreased expression of pro‐inflammatory cytokines (IL‐1β and TNF‐α) (Figure 5C). Together, these results demonstrate that GDF11 promotes functional recovery and remyelination following cuprizone‐induced injury, likely through coordinated enhancement of OL maturation and suppression of neuroinflammation.

### 3.4 GDF11 Promotes Remyelination and Functional Recovery in the EAE Model of MS

**FIGURE 6:**
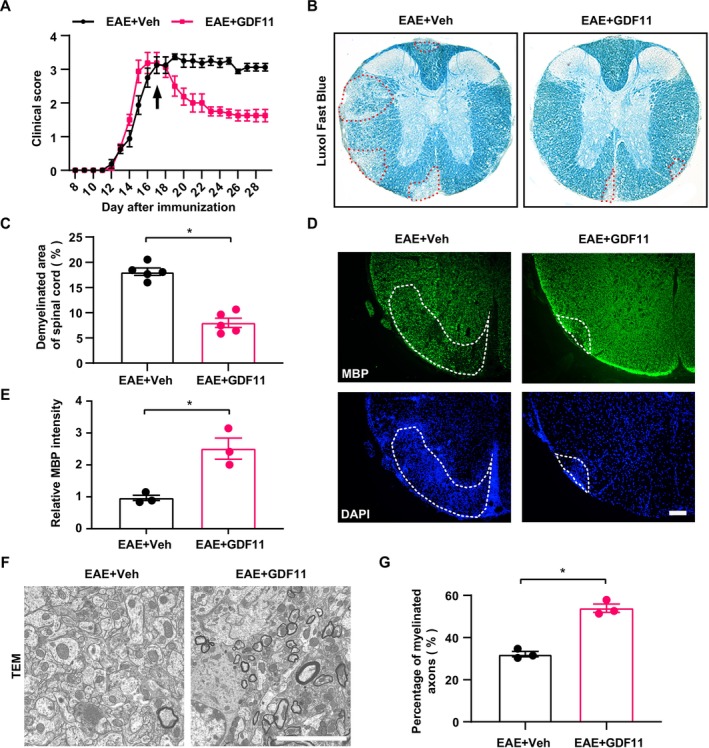
Therapeutic efficacy of GDF11 in the EAE mouse model. (A) Clinical score of MOG_35‐55_‐induced EAE mice treated daily with GDF11 or Veh beginning at peak disease (black arrows, D17) (*n* = 12 mice per group). (B) Representative images of Luxol fast blue staining in spinal cord sections from GDF11‐ or Veh‐treated EAE mice. Demyelinated areas are outlined by dashed lines. (C) Quantification of demyelinated area in the spinal cord of GDF11‐ or Veh‐treated EAE mice (*n* = 5 mice per group). (D) Representative immunofluorescence staining of MBP in the spinal cord of GDF11‐ or Veh‐treated EAE mice. Scale bar, 100 μm. (E) Quantification of MBP fluorescence intensity in the spinal cord of GDF11‐ or Veh‐treated EAE mice (*n* = 3 mice per group). (F) Transmission electron microscopy (TEM) images of the ventral white matter of the spinal cord from GDF11‐ or Veh‐treated EAE mice. Scale bar, 2 μm. (G) Percentage of myelinated axons in the ventral white matter of the spinal cord (*n* = 3 mice per group). Data are presented as mean ± SEM. *p < 0.05.

Finally, we evaluated the therapeutic efficacy of GDF11 in the MOG_35_‐_55_‐induced EAE model, which recapitulates key features of MS, including autoimmune‐mediated demyelination and BBB disruption. Beginning at day 17 post‐immunization, corresponding to peak disease severity, mice received daily intraperitoneal injections of GDF11 or Veh for 2 weeks (Figure 6A). GDF11‐treated mice exhibited pronounced functional recovery, with most animals regaining partial or full use of one or both hind limbs, reaching a mean clinical score of approximately 1 (Figure 6A). In contrast, Veh‐treated mice developed persistent severe paralysis (scores 2–3). Histological analyses corroborated these functional improvements. Luxol fast blue staining revealed a significant reduction in demyelinated lesion area in the spinal cords of GDF11‐treated mice (Figure 6B,C), while immunohistochemistry demonstrated robust restoration of MBP expression (Figure 6D,E). Ultrastructural analysis further confirmed a higher proportion of myelinated axons and characteristic thinly myelinated fibers indicative of active remyelination in GDF11‐treated animals (Figure 6F,G). Collectively, these findings demonstrate that peripheral GDF11 administration promotes substantial functional recovery and structural remyelination in autoimmune demyelination, underscoring its therapeutic potential for MS.

## 4 Discussion

Efficient remyelination is essential for functional recovery and long‐term neuroprotection in multiple sclerosis and other demyelinating disorders. However, endogenous remyelination often fails, despite the persistence of oligodendrocyte progenitor cells within lesions. In the present study, we identify GDF11 as a previously unrecognized circulating regulator of oligodendrocyte differentiation, CNS myelination, and remyelination. Through complementary in vitro and in vivo approaches, we demonstrate that systemic GDF11 directly promotes OPC maturation, enhances developmental myelination, and accelerates structural and functional recovery following both toxic and immune‐mediated demyelination.

### 4.1 GDF11 Directly Drives Oligodendroglial Lineage Progression

A key finding of this study is that GDF11 exerts a direct, cell‐autonomous effect on OPC differentiation. In purified OPC cultures, GDF11 robustly promoted the transition from immature progenitors to mature, MBP‐expressing oligodendrocytes without altering cell proliferation or survival. This selective effect on lineage maturation distinguishes GDF11 from mitogenic factors such as PDGF and bFGF, which primarily regulate OPC expansion [7, 8, 9, 10]. Moreover, GDF11 enhanced the morphological complexity of mature oligodendrocytes, a hallmark of functional myelin‐forming capacity. These findings place GDF11 among a growing but still limited group of factors that preferentially drive OL maturation rather than progenitor proliferation. Mechanistically, GDF11 signals through ALK4/5/7 receptors to activate Smad2/3‐dependent pathways, which have previously been implicated in OL differentiation and myelin gene expression [16, 17, 34]. Although direct pathway dissection was beyond the scope of the current study, our data are consistent with the notion that GDF11 engages conserved TGF‐β family signaling cascades to promote oligodendroglial maturation. Future studies employing receptor‐specific or pathway‐specific genetic and pharmacological approaches will be necessary to define the precise downstream mechanisms.

### 4.2 Systemic GDF11 Enhances CNS Myelination Despite an Intact BBB

An unexpected and conceptually important observation is that peripherally administered GDF11 enhanced developmental myelination in neonatal mice, even under physiological conditions in which the blood–brain barrier is largely intact. This finding challenges the prevailing assumption that circulating factors exert limited influence on CNS myelination in the absence of overt BBB disruption. Several non‐mutually exclusive mechanisms may account for this effect. First, GDF11 may cross the BBB at low but biologically effective levels, particularly during early postnatal development when BBB properties are still maturing [26, 35]. Alternatively, GDF11 may act indirectly by modulating endothelial cells, pericytes, or other components of the neurovascular unit, thereby influencing oligodendroglial differentiation through secondary signaling mechanisms. Consistent with this possibility, GDF11 has been shown to regulate vascular remodeling and endothelial function in other contexts [22, 26, 36, 37, 38]. These findings suggest that CNS myelination may be more sensitive to systemic cues than previously appreciated.

Notably, although GDF11 treatment from P2 to P16 significantly increased myelination at P16, this effect was no longer detectable at 6 weeks of age (P42). These results indicate that GDF11 accelerates the kinetics of developmental myelination without altering the final myelin content in adulthood—a pattern consistent with other promyelinating factors such as thyroid hormone, which advances the timing of myelination but does not change ultimate myelin abundance [39]. Thus, the effect of GDF11 is not a permanent increase but rather a temporal acceleration of myelination.

### 4.3 Dual Actions of GDF11 in Remyelination and Neuroinflammation

In both cuprizone and EAE models, GDF11 treatment was associated with reduced microglial activation and decreased expression of pro‐inflammatory cytokines. While our in vitro data demonstrate a direct effect of GDF11 on OPC differentiation, the in vivo remyelinating effects are likely amplified by a more permissive inflammatory environment. Whether these immunomodulatory effects arise from direct actions of GDF11 on microglia or indirectly through altered oligodendrocyte–microglia interactions remains to be determined. Consistent with the former possibility, previous studies have demonstrated that GDF11 can promote an anti‐inflammatory microglial phenotype via signaling pathways involving TGF‐βR1/Smad2/NF‐κB and p38 MAPK, leading to reduced neuroinflammation and improved functional outcomes in models of neuropathic pain, spinal cord injury, and cognitive impairment [40, 41, 42]. Furthermore, GDF11 has also been reported to enhance hippocampal neurogenesis and cognitive performance in diabetic mice by suppressing neuroinflammatory responses, underscoring its broader capacity to modulate the neuroimmune environment [43]. Taken together, these findings support a model in which GDF11 biases microglia toward a pro‐repair state that is permissive for remyelination. Importantly, such immunomodulatory effects occur in parallel with enhanced oligodendrocyte differentiation, suggesting that GDF11 promotes CNS repair through coordinated, multi‐cellular mechanisms rather than through isolated actions on a single cell type.

### 4.4 Therapeutic Implications for MS and Demyelinating Diseases

The robust efficacy of GDF11 in the EAE model highlights its translational potential for immune‐mediated demyelinating diseases. Unlike current MS therapies, which primarily target immune activation [44], GDF11 directly engages endogenous repair mechanisms. The ability of peripherally administered GDF11 to promote remyelination and functional recovery positions it as a promising candidate for combination therapies aimed at both suppressing inflammation and restoring myelin integrity. Nevertheless, several limitations should be acknowledged. The long‐term safety, optimal dosing, and temporal window for GDF11 treatment remain to be determined. Additionally, whether GDF11 exerts similar effects in chronic demyelinating settings or in aged animals—where remyelination capacity is markedly reduced—will be critical questions for future studies.

## 5 Conclusion

In summary, our study identifies GDF11 as a circulating factor that promotes oligodendrocyte differentiation, enhances CNS myelination, and accelerates remyelination and functional recovery following demyelinating injury. These findings expand our understanding of how systemic signals regulate myelin dynamics and reveal GDF11 as a potential therapeutic agent for promoting myelin repair in MS and related disorders.

## Author Contributions

Z.Z., W.C., T.L., and Y.C. designed research and interpreted data; Z.Z. performed experiments with assistance from M.Y., W.H. and T.L.; Z.Z. and M.Y. performed microscopy, data gathering, and imaging. Y.C. contributed to manuscript editing; Z.Z., T.L, and W.C. wrote the manuscript.

## Funding

This study was supported by the National Natural Science Foundation of China (No. 81660278), Yunnan Provincial Science and Technology Department–Applied Basic Research Joint Special Funds of Kunming Medical University (No. 202301AY070001‐089), Talent Program of Yunnan Province (KH‐2025‐XDYC‐YLWS‐02), and Medical Joint Special Project of Kunming University of Science and Technology—the First People's Hospital of Yunnan Province (No. KUST‐KH2022015Y).

## Ethics Statement

All experiments were performed in accordance with the Guide for the Care and Use of Laboratory Animals and approved by the Animal Care and Ethics Committee of Kunming University of Science and Technology (Approval number: PZWH K2023‐0031).

## Conflicts of Interest

The authors declare no conflicts of interest.

## Data Availability

The data that support the findings of this study are available from the corresponding author upon reasonable request.

## References

[1] A. J. Thompson , S. E. Baranzini , J. Geurts , B. Hemmer , and O. Ciccarelli , “Multiple Sclerosis,” Lancet 391 (2018): 1622–1636. 29576504 10.1016/S0140-6736(18)30481-1

[2] T. Ziemssen , T. Derfuss , N. de Stefano , et al., “Optimizing Treatment Success in Multiple Sclerosis,” Journal of Neurology 263 (2016): 1053–1065. 26705122 10.1007/s00415-015-7986-y PMC4893374

[3] H. N. Lemus , A. E. Warrington , and M. Rodriguez , “Multiple Sclerosis: Mechanisms of Disease and Strategies for Myelin and Axonal Repair,” Neurologic Clinics 36 (2018): 1–11. 29157392 10.1016/j.ncl.2017.08.002 PMC7125639

[4] S. L. Hauser and B. A. C. Cree , “Treatment of Multiple Sclerosis: A Review,” American Journal of Medicine 133 (2020): 1380–1390.e1382. 32682869 10.1016/j.amjmed.2020.05.049 PMC7704606

[5] A. Chang , W. W. Tourtellotte , R. Rudick , and B. D. Trapp , “Premyelinating Oligodendrocytes in Chronic Lesions of Multiple Sclerosis,” New England Journal of Medicine 346 (2002): 165–173. 11796850 10.1056/NEJMoa010994

[6] M. Amin and C. M. Hersh , “Updates and Advances in Multiple Sclerosis Neurotherapeutics,” Neurodegenerative Disease Management 13 (2023): 47–70. 36314777 10.2217/nmt-2021-0058 PMC10072078

[7] A. R. Calver , A. C. Hall , W. P. Yu , et al., “Oligodendrocyte Population Dynamics and the Role of PDGF In Vivo,” Neuron 20 (1998): 869–882. 9620692 10.1016/s0896-6273(00)80469-9

[8] A. Sherafat , F. Pfeiffer , A. M. Reiss , W. M. Wood , and A. Nishiyama , “Microglial Neuropilin‐1 Promotes Oligodendrocyte Expansion During Development and Remyelination by Trans‐Activating Platelet‐Derived Growth Factor Receptor,” Nature Communications 12 (2021): 2265. 10.1038/s41467-021-22532-2 PMC8050320 33859199

[9] M. Maiworm , “The Relevance of BDNF for Neuroprotection and Neuroplasticity in Multiple Sclerosis,” Frontiers in Neurology 15 (2024): 1385042. 39148705 10.3389/fneur.2024.1385042 PMC11325594

[10] M. Furusho , A. J. Roulois , R. J. Franklin , and R. Bansal , “Fibroblast Growth Factor Signaling in Oligodendrocyte‐Lineage Cells Facilitates Recovery of Chronically Demyelinated Lesions but Is Redundant in Acute Lesions,” Glia 63 (2015): 1714–1728. 25913734 10.1002/glia.22838 PMC4534313

[11] C. Takahashi , R. Muramatsu , H. Fujimura , H. Mochizuki , and T. Yamashita , “Prostacyclin Promotes Oligodendrocyte Precursor Recruitment and Remyelination After Spinal Cord Demyelination,” Cell Death & Disease 4 (2013): e795. 24030147 10.1038/cddis.2013.335 PMC3789193

[12] A. A. Abulaban , H. M. Al‐Kuraishy , A. I. Al‐Gareeb , et al., “The Possible Role of Metformin and Fibroblast Growth Factor‐21 in Multiple Sclerosis Neuropathology: Birds of a Feather Flock Together,” European Journal of Neuroscience 61 (2025): e70067. 40172524 10.1111/ejn.70067 PMC11963988

[13] W. Li , C. Berlinicke , Y. Huang , et al., “High‐Throughput Screening for Myelination Promoting Compounds Using Human Stem Cell‐Derived Oligodendrocyte Progenitor Cells,” iScience 26 (2023): 106156. 36852281 10.1016/j.isci.2023.106156 PMC9958491

[14] A. Esmaeilzadeh , V. Mohammadi , and R. Elahi , “Transforming Growth Factor Beta (TGF‐Beta) Pathway in the Immunopathogenesis of Multiple Sclerosis (MS); Molecular Approaches,” Molecular Biology Reports 50 (2023): 6121–6131. 37204543 10.1007/s11033-023-08419-z

[15] R. K. Holloway , G. Ireland , G. Sullivan , et al., “Microglial Inflammasome Activation Drives Developmental White Matter Injury,” Glia 69 (2021): 1268–1280. 33417729 10.1002/glia.23963 PMC8607465

[16] J. Palazuelos , M. Klingener , and A. Aguirre , “TGFbeta Signaling Regulates the Timing of CNS Myelination by Modulating Oligodendrocyte Progenitor Cell Cycle Exit Through SMAD3/4/FoxO1/Sp1,” Journal of Neuroscience 34 (2014): 7917–7930. 24899714 10.1523/JNEUROSCI.0363-14.2014 PMC4044250

[17] D. J. Dutta , A. Zameer , J. N. Mariani , et al., “Combinatorial Actions of Tgfbeta and Activin Ligands Promote Oligodendrocyte Development and CNS Myelination,” Development 141 (2014): 2414–2428. 24917498 10.1242/dev.106492 PMC4050697

[18] V. E. Miron , A. Boyd , J. W. Zhao , et al., “M2 Microglia and Macrophages Drive Oligodendrocyte Differentiation During CNS Remyelination,” Nature Neuroscience 16 (2013): 1211–1218. 23872599 10.1038/nn.3469 PMC3977045

[19] P. Habibi , K. Falamarzi , N. D. Ebrahimi , M. Zarei , M. Malekpour , and N. Azarpira , “GDF11: An Emerging Therapeutic Target for Liver Diseases and Fibrosis,” Journal of Cellular and Molecular Medicine 28 (2024): e18140. 38494851 10.1111/jcmm.18140 PMC10945076

[20] C. Moigneu , S. Abdellaoui , M. Ramos‐Brossier , et al., “Systemic GDF11 Attenuates Depression‐Like Phenotype in Aged Mice via Stimulation of Neuronal Autophagy,” Nature Aging 3 (2023): 213–228. 37118117 10.1038/s43587-022-00352-3 PMC10154197

[21] F. S. Loffredo , M. L. Steinhauser , S. M. Jay , et al., “Growth Differentiation Factor 11 Is a Circulating Factor That Reverses Age‐Related Cardiac Hypertrophy,” Cell 153 (2013): 828–839. 23663781 10.1016/j.cell.2013.04.015 PMC3677132

[22] L. Katsimpardi , N. K. Litterman , P. A. Schein , et al., “Vascular and Neurogenic Rejuvenation of the Aging Mouse Brain by Young Systemic Factors,” Science 344 (2014): 630–634. 24797482 10.1126/science.1251141 PMC4123747

[23] M. Sinha , Y. C. Jang , J. Oh , et al., “Restoring Systemic GDF11 Levels Reverses Age‐Related Dysfunction in Mouse Skeletal Muscle,” Science 344 (2014): 649–652. 24797481 10.1126/science.1251152 PMC4104429

[24] W. Machelak , A. Szczepaniak , D. Jacenik , and M. Zielinska , “The Role of GDF11 During Inflammation—An Overview,” Life Sciences 322 (2023): 121650. 37011872 10.1016/j.lfs.2023.121650

[25] J. Duran , M. F. Troncoso , D. Lagos , S. Ramos , G. Marin , and M. Estrada , “GDF11 Modulates Ca(2+)‐Dependent Smad2/3 Signaling to Prevent Cardiomyocyte Hypertrophy,” International Journal of Molecular Sciences 19 (2018): 1508. 29783655 10.3390/ijms19051508 PMC5983757

[26] C. Ozek , R. C. Krolewski , S. M. Buchanan , and L. L. Rubin , “Growth Differentiation Factor 11 Treatment Leads to Neuronal and Vascular Improvements in the Hippocampus of Aged Mice,” Scientific Reports 8 (2018): 17293. 30470794 10.1038/s41598-018-35716-6 PMC6251885

[27] R. G. Walker , T. Poggioli , L. Katsimpardi , et al., “Biochemistry and Biology of GDF11 and Myostatin: Similarities, Differences, and Questions for Future Investigation,” Circulation Research 118 (2016): 1125–1142. 27034275 10.1161/CIRCRESAHA.116.308391 PMC4818972

[28] B. Emery and J. C. Dugas , “Purification of Oligodendrocyte Lineage Cells From Mouse Cortices by Immunopanning,” Cold Spring Harbor Protocols 2013 (2013): 854–868. 24003195 10.1101/pdb.prot073973

[29] E. G. Hughes , S. H. Kang , M. Fukaya , and D. E. Bergles , “Oligodendrocyte Progenitors Balance Growth With Self‐Repulsion to Achieve Homeostasis in the Adult Brain,” Nature Neuroscience 16 (2013): 668–676. 23624515 10.1038/nn.3390 PMC3807738

[30] L. P. Fang , C. H. Lin , Y. Medlej , et al., “Oligodendrocyte Precursor Cells Facilitate Neuronal Lysosome Release,” Nature Communications 16 (2025): 1175. 10.1038/s41467-025-56484-8 PMC11782495 39885146

[31] A. J. Steelman , J. P. Thompson , and J. Li , “Demyelination and Remyelination in Anatomically Distinct Regions of the Corpus Callosum Following Cuprizone Intoxication,” Neuroscience Research 72 (2012): 32–42. 22015947 10.1016/j.neures.2011.10.002 PMC3230728

[32] P. Morell , C. V. Barrett , J. L. Mason , et al., “Gene Expression in Brain During Cuprizone‐Induced Demyelination and Remyelination,” Molecular and Cellular Neurosciences 12 (1998): 220–227. 9828087 10.1006/mcne.1998.0715

[33] M. Lindner , S. Heine , K. Haastert , et al., “Sequential Myelin Protein Expression During Remyelination Reveals Fast and Efficient Repair After Central Nervous System Demyelination,” Neuropathology and Applied Neurobiology 34 (2008): 105–114. 17961136 10.1111/j.1365-2990.2007.00879.x

[34] R. G. Walker , M. Czepnik , E. J. Goebel , et al., “Structural Basis for Potency Differences Between GDF8 and GDF11,” BMC Biology 15 (2017): 19. 28257634 10.1186/s12915-017-0350-1 PMC5336696

[35] H. H. Su , J. C. Yen , J. M. Liao , et al., “In Situ Slow‐Release Recombinant Growth Differentiation Factor 11 Exhibits Therapeutic Efficacy in Ischemic Stroke,” Biomedicine & Pharmacotherapy 144 (2021): 112290. 34673423 10.1016/j.biopha.2021.112290

[36] L. Katsimpardi , N. Kuperwasser , C. Camus , et al., “Systemic GDF11 Stimulates the Secretion of Adiponectin and Induces a Calorie Restriction‐Like Phenotype in Aged Mice,” Aging Cell 19 (2020): e13038. 31637864 10.1111/acel.13038 PMC6974718

[37] L. Piccio , C. Cantoni , J. G. Henderson , et al., “Lack of Adiponectin Leads to Increased Lymphocyte Activation and Increased Disease Severity in a Mouse Model of Multiple Sclerosis,” European Journal of Immunology 43 (2013): 2089–2100. 23640763 10.1002/eji.201242836 PMC3901539

[38] Y. Zhao , W. Zhu , T. Wan , et al., “Vascular Endothelium Deploys Caveolin‐1 to Regulate Oligodendrogenesis After Chronic Cerebral Ischemia in Mice,” Nature Communications 13 (2022): 6813. 10.1038/s41467-022-34293-7 PMC9649811 36357389

[39] S. N. Walters and P. Morell , “Effects of Altered Thyroid States on Myelinogenesis,” Journal of Neurochemistry 36 (1981): 1792–1801. 7241137 10.1111/j.1471-4159.1981.tb00433.x

[40] T. Liu and L. Zhang , “GDF11 Mitigates Neuropathic Pain via Regulation of Microglial Polarization and Neuroinflammation Through TGF‐betaR1/SMAD2/NF‐kappaB Pathway in Male Mice,” Journal of Neuroimmune Pharmacology 20 (2025): 20. 39939465 10.1007/s11481-025-10172-y

[41] X. Zhao , R. Qin , G. Li , et al., “GDF11 Regulates M1 and M2 Polarization of BV2 Microglial Cells via p38 MAPK Signaling Pathway,” Molecular Neurobiology 62 (2025): 9290–9305. 40100492 10.1007/s12035-025-04837-1

[42] Z. Wang , Y. Zhang , W. Liu , et al., “GDF11 Alleviates Spinal Cord Injury in Rats by Modulating Microglia Polarization Through Smad2/3 and MAPK/NFkappaB Signaling Pathways,” International Immunopharmacology 163 (2025): 115197. 40674843 10.1016/j.intimp.2025.115197

[43] Y. Xing , X. Ma , R. Zhai , W. Chen , and H. Yan , “GDF11 Improves Hippocampal Neurogenesis and Cognitive Abilities in Diabetic Mice by Reducing Neural Inflammation,” Brain, Behavior, and Immunity 120 (2024): 21–31. 38777287 10.1016/j.bbi.2024.05.024

[44] S. Faissner , J. R. Plemel , R. Gold , and V. W. Yong , “Progressive Multiple Sclerosis: From Pathophysiology to Therapeutic Strategies,” Nature Reviews. Drug Discovery 18 (2019): 905–922. 31399729 10.1038/s41573-019-0035-2

